# An Enhanced Food Digestion Algorithm for Mobile Sensor Localization

**DOI:** 10.3390/s23177508

**Published:** 2023-08-29

**Authors:** Shu-Chuan Chu, Zhi-Yuan Shao, Ning Zhong, Geng-Geng Liu, Jeng-Shyang Pan

**Affiliations:** 1 College of Computer Science and Engineering, Shandong University of Science and Technology, Qingdao 266590, China; scchu0803@sdust.edu.cn (S.-C.C.); 202082060043@sdust.edu.cn (Z.-Y.S.); 2 College of Science and Engineering, Flinders University, 1284 South Road, Tonsley, SA 5042, Australia; 3 Department of Life Science and Informatics, Maebashi Institute of Technology, Maebashi 371-0816, Japan; zhong@maebashi-it.ac.jp; 4 International WIC Institute, Beijing University of Technology, Beijing 100124, China; 5 Beijing International Collaboration Base on Brain Informatics and Wisdom Services, Beijing 100124, China; 6 College of Mathematics and Computer Science, Fuzhou University, Fuzhou 350116, China; liugenggeng@fzu.edu.cn; 7 Department of Information Management, Chaoyang University of Technology, Taichung 41349, Taiwan

**Keywords:** food digestion algorithm, parallel strategy, compact strategy, mobile sensors, Monte Carlo Localization

## Abstract

Mobile sensors can extend the range of monitoring and overcome static sensors’ limitations and are increasingly used in real-life applications. Since there can be significant errors in mobile sensor localization using the Monte Carlo Localization (MCL), this paper improves the food digestion algorithm (FDA). This paper applies the improved algorithm to the mobile sensor localization problem to reduce localization errors and improve localization accuracy. Firstly, this paper proposes three inter-group communication strategies to speed up the convergence of the algorithm based on the topology that exists between groups. Finally, the improved algorithm is applied to the mobile sensor localization problem, reducing the localization error and achieving good localization results.

## 1. Introduction

Metaheuristic algorithms play a huge role in many fields, such as UAV [1,2,3], digital watermarking [4], engineering design [5], unit commitment [6], image processing [7,8,9], intrusion detection systems [10], feature selection [11], multi-robot exploration [12], wireless sensor networks [13,14,15], scheduling problems [16,17], etc.

Although metaheuristics are excellent at solving problems with real-world applications, they are not a panacea, and, as mentioned in the No Free Lunch Theorem [18], each optimization algorithm may be good at solving different problems. Therefore, researchers are constantly exploring new optimization algorithms. For example, Holland proposed the Genetic Algorithm (GA) in 1975 based on Darwinian evolutionary theory [19]. Dorigo et al. proposed the Ant Colony Optimization (ACO) in 1992 [20]. Storn et al. proposed Differential Evolution (DE) in 1995 [21]. Kennedy and Eberhart proposed the Particle Swarm Optimization (PSO) algorithm in 1995 [22]. Karaboga et al. proposed the Artificial Bee Colony algorithm (ABC) in 2005 [23]. Yang et al. proposed the Cuckoo Search (CS) in 2009 [24]. Rashedi et al. proposed the Gravitational Search Algorithm in 2009 [25]. Yang et al. proposed the Bat Algorithm (BA) in 2010 [26]. Mirjalili et al. proposed the Grey Wolf Optimizer (GWO) in 2014 [27]. Mirjalili et al. proposed the Sine Cosine Algorithm (SCA) in 2016 [28]. Abualigah et al. proposed the Aquila Optimizer (AO) in 2021 [29]. Song et al. proposed the Phasmatodea Population Evolution algorithm (PPE) in 2021 [30]. Pan et al. proposed the Gannet Optimization Algorithm (GOA) in 2022 [31].

Numerous researchers have dedicated their efforts to enhancing the performance of metaheuristic algorithms. Among the various approaches, parallel and compact strategies have gained significant attention due to their simplicity and effectiveness. The parallel strategy emphasizes the grouping of populations, facilitating the exchange of information between groups to accelerate the algorithm’s convergence and enhance its ability to discover optimal solutions accurately. On the other hand, the compact strategy involves mapping the population onto a probabilistic model and performing operations on the entire population through manipulations of this model. This approach offers notable benefits such as reduced computational time and memory usage. In this study, we propose a novel approach that combines both parallel and compact strategies to enhance the performance of the food digestion algorithm. We expect that this integrated methodology will effectively enhance the algorithm’s ability to seek optimal solutions in the optimization process, leading to improved outcomes.

Numerous researchers have combined these two strategies to improve metaheuristic algorithms. In reference [32], the authors combine the parallel and compact strategies to enhance DE and utilize the enhanced algorithm for image segmentation, yielding superior outcomes. In reference [33], the authors initially introduce six enhancements to the compact strategy CS, subsequently selecting the algorithm with the most favorable results and incorporating the parallel strategy. Ultimately, the authors apply the improved algorithm to underwater robot path planning, which yields promising results.

Wireless sensor networks (WSNs) are self-organized communication systems consisting of multiple nodes that enable the monitoring of specific areas through multi-hop communication. In a static WSN, the nodes are randomly distributed and their locations remain fixed once determined. However, in practical environments, mobile sensor nodes are in greater demand. For instance, in target tracking applications, real-time positioning of moving targets is essential [34,35]. The mobility of sensor nodes allows for an extended monitoring range, overcoming coverage gaps that may occur due to the failure of static nodes. Furthermore, the movement of nodes enables the network to discover and observe events more effectively, while also enhancing the communication quality among the sensor nodes [36]. Despite the importance of mobile node localization, there is a relative scarcity of research in this area. Most localization methods developed for static sensor nodes are unsuitable for the mobile sensor localization problem, making the study of mobile sensor localization a current research focal point [37]. Additionally, the study of outdoor mobile sensors holds particular significance due to the complex and ever-changing nature of the outdoor environment.

Based on the above reasons, this paper uses parallel and compact strategies to improve the food digestion algorithm and apply it to the outdoor mobile sensor localization problem. Section 2 mainly introduces the food digestion algorithm and mobile sensor localization techniques. Section 3 mainly introduces the implementation of the Parallel Compact Food Digestion Algorithm (PCFDA). Section 4 tests the performance of PCFDA. Section 5 uses PCFDA to optimize the error in mobile sensor localization. Section 6 gives the conclusion of this paper.

## 2. Related Works

This section mainly introduces the food digestion algorithm and the mobile sensor localization problem.

### 2.1. Food Digestion Algorithm

The food digestion algorithm mainly covers the process of food digestion in the mouth, stomach, and small intestine. This section describes the modeling processes in these three sites in detail.

#### 2.1.1. Digestion in the Oral Cavity

The digestion of food in the mouth involves both physical and chemical digestion. The process of physical digestion mainly consists of the action of forces, which are represented as follows:

(1) $$F1=2*(-\arctan\left(iter*a/Max_{-}iter-a\right))$$

(2) $$F1_{-}d=2*F1*rand-F1$$

F1 denotes the force on the food in the mouth, iter denotes the current number of iterations, $Max_{-}iter$ denotes the maximum number of iterations, and *a* is used to adjust the size of F1, which has a value of 1.5574. $F1_{-}d$ denotes forces with different sizes and directions, where rand is a random value in the range [0, 1].

The chemical digestion of food in the mouth is dominated by the digestion of starch by salivary amylase, and, considering the effect of substrate concentration on the enzymatic reaction, the modeling process is as follows:

(3) $$\begin{array}{c} Em^{\left(i\right)}=\left\{\begin{array}{ccc} 1 & if & r(i)>(D/2),r=randperm(D)\\ 0 & \; & else \end{array}\right.i=1,2\ldots D \end{array}$$

(4) $$V=\left(V_{\max}*S\right)/\left(K_{m}+S\right)$$

(5) $$S=\sin\left(rand*\pi \right)$$

Em denotes the enzyme in the oral cavity, randomly setting half of the dimension values to 0 and the other half to 1. r=randperm(D) denotes that the values of the *D* dimensions are randomly scrambled. Equation (4) is the Mie equation, which reflects the relationship between substrate concentration and reaction rate [38]. *V* represents the rate of the enzymatic reaction, $V_{\max}$ represents the maximum reaction rate, and its value is 2. *S* represents the substrate concentration, and we express it as a sine function, represented by Equation (5), where rand is a number in the range [0,1]. π represents the mathematical constant pi. $K_{m}$ is a characteristic constant of the enzyme, and in the oral cavity, the value of $K_{m}$ is 0.8. Therefore, the particle update equation in the oral cavity is as follows:

(6) $$\begin{array}{cc} {Food}_{i}^{t+1}= & {Food}_{k}^{t}+F1_{-}d*\left(C1*{Best}_{-}p-{Food}_{i}^{t}\right)+C1*Em*\left({Food}_{i}^{t}-C2*{Food}_{R}^{t}\right)*V \end{array}$$

(7) k=randi(N)

(8) $$R=randi\left(ceil\left(\left(N/3\right)*e^{-iter*b/{Max}_{-}iter}\right)\right)$$

${Food}_{i}^{t+1}$ denotes the ith particle at generation t+1. ${Food}_{k}^{t}$ denotes the kth particle at generation *t*. *k* is a randomly selected particle from among *N* particles. ${Food}_{R}^{t}$ denotes the *R*th particle of the tth generation, and *R* is chosen as shown in Equation (8). *b* is a constant which has a value of 1.5. ${Food}_{i}^{t}$ denotes the ith particle of the tth generation. ${Best}_{-}p$ represents the global optimal value. C1 and C2 are two random numbers that change with the number of iterations. ceil denotes rounding to positive infinity, and randi is a random rounding function.

#### 2.1.2. Digestion in the Stomach

The digestion of food in the stomach also involves two processes: physical and chemical digestion. Physical digestion is primarily governed by the forces generated by the contraction and diastole of the stomach as well as peristalsis. The forces are expressed as follows:

(9) $$F2=2*(1-\left(iter/Max_{-}iter\right))$$

(10) $$F2_{-}d=2*F2*rand-F2$$

F2 represents the force on the food in the stomach. $F2_{-}d$ represents a directed force, which takes values in the range [−2, 2]. The chemical digestion modeling process in the stomach is similar to that in the oral cavity. The difference is that different enzymes Em and different characteristic constants $K_{m}$ are selected for each iteration. In the stomach, the value of $K_{m}$ is 0.9. Therefore, the particle update equation in the stomach is as follows:

(11) $$\begin{array}{cc} {Food}_{i}^{t+1}= & {Food}_{m}^{t+1}+F2_{-}d*\left({Food}_{i}^{t}-C1*{Food}_{m}^{t+1}\right)+C1*Em*\left({Food}_{m}^{t+1}-Mean\right)*V \end{array}$$

(12) $$Mean=\frac{1}{N}\left(\sum_{i=1}^{N/3}{Food}_{i}^{t+1}+\sum_{i=N/3}^{N}{Food}_{i}^{t}\right)$$

(13) $$Food_{m}^{t+1}=\left\{\begin{array}{c} Food_{m}^{t+1}\\ Best_{-}p+\left(C1*{Best}_{-}p-{Food}_{m}^{t+1}\right) \end{array}\;\right.$$

$Food_{m}^{t+1}$ selects particles according to Equation (13). If the optimal fitness value of the first one-third of the updated particles is less than the global optimum, then we select this particle. Otherwise, we perturb the globally optimal particle and select the perturbed particle. Therefore, its selection condition is $if\min\left(fitness_{i}^{t+1}\right)<Best_{-}p,i\in \left(1,N/3\right)$. Mean is calculated according to Equation (12).

#### 2.1.3. Digestion in the Small Intestine

The digestion of food in the small intestine also involves two processes: physical and chemical digestion. Physical digestion is primarily governed by forces generated by peristalsis of the small intestine, which is expressed as follows:

(14) $$F3=2*\left(-\arctan\left(iter*a/Max_{-}iter\right)+a_{1}\right)$$

(15) $$F3_{-}d=2*F3*rand-F3$$

F3 represents the force on the food in the small intestine. *a* is a constant that has a value of 1.5574. $a_{1}$ is used to regulate the magnitude of the force, which has a value of 1. $F3_{-}d$ represents a directed force, which is a random value in the range [−2, 2]. Thus, the equation for particles updated in the small intestine is as in Equation (16).

(16) $${Food}_{i}^{t+1}={Best}_{-}p+F3_{-}d*\left({Food}_{i}^{t}-C1*{Food}_{n}^{t+1}\right)+C1*Em*Levy\left(D\right)*V$$

(17) $$Food_{n}^{t+1}=\left\{\begin{array}{c} Food_{n}^{t+1}\\ Best_{-}p+\left(C1*{Best}_{-}p-{Food}_{n}^{t+1}\right) \end{array}\right.$$

The judgment condition for $Food_{n}^{t+1}$ is $if\min\left({fitness}_{i}^{t+1}\right)<Best_{-}p,i\in \left(N/3,N*\left(2/3\right)\right)$, which is calculated from Equation (17). Levy(D) denotes Lévy flight, which is calculated as follows:

(18) $$Levy\left(D\right)=0.01*\frac{\mu *\delta }{{\left|v\right|}^{\frac{1}{\beta }}}$$

(19) $$\delta ={\left(\frac{\Gamma \left(1+\beta \right)*sin\left(\frac{\beta *\pi }{2}\right)}{\Gamma \left(\frac{1+\beta }{2}\right)*\beta *2^{\frac{\beta -1}{2}}}\right)}^{\frac{1}{\beta }}$$

μ and δ are random numbers in the range [0, 1], and β is a constant whose value is 1.5.

The food digestion algorithm simulates the process of food digestion in the three main digestive sites in the human body to construct the particle optimization process. In the oral cavity, particles always follow a random particle to update their positions, which promotes the diversity of particles. As the number of iterations increases, particles gradually select particles with better fitness values to update their positions. This selection enhances population diversity in the early iterations and facilitates rapid convergence in later stages.

In the stomach, particles follow the optimal particles from the previous site or particles after perturbation to update their positions. This accelerates the convergence process. Additionally, particles follow the average particles to update their positions, promoting particle diversity and preventing them from getting trapped in local optima.

In the small intestine, particles update their positions after the global optimum, enabling quick convergence. Furthermore, particles update their positions using the Lévy flight strategy, which helps avoid falling into local optima.

Algorithm 1 provides a detailed description of the FDA.
**Algorithm 1** Food Digestion Algorithm**Output:** Population size *N*; Dimension *D*; Maximum number of iterations $Max_{-}iter$; Lower boundary lb; Upper boundary ub;**Input:** Global optimal position $Best_{-}p$, Global optimal fitness value $Best_{-}f$;1:Initialize populations and calculate their fitness values;2:Record the optimum global position $Best_{-}p$;3:Initialize the parameters $a,b,a_{1},V_{max},K_{m}$;4:**while** $iter<Max_{-}iter$ **do**5:    Backup the initialized populations and their fitness values;6:    Calculate the values of F1, F2, and F3 according to Equations (1), (9) and (14);7:    Calculate the value of *R* according to Equation (8);8:    Calculate the values of C1 and C2;9:    **for** i=1:N **do**10:        Calculate the values of Em and *S* according to Equations (3) and (5);11:        **if** i<=N/3 **then**12:           Calculate the values of $F1_{-}d$ and *V* according to Equations (2) and (4);13:           Update the particle according to Equation (6);14:           Calculate the fitness value of the particle;15:           **if** i==N/3 **then**16:               Find the minimum fitness value in the oral cavity $fitness_{m}$;17:               Update the particle according to Equation (13);18:           **end if**19:        **end if**20:        **if** i>N/3 and i<=2∗N/3 **then**21:           Calculate the values of $F2_{-}d$, *V*, and Mean according to Equations (4), (10) and (12);22:           Update the particle according to Equation (11);23:           Calculate the fitness value of the particle;24:           **if** i==2∗N/3 **then**25:               Find the minimum fitness value in the stomach $fitness_{n}$;26:               Update the particle according to Equation (17);27:           **end if**28:        **end if**29:        **if** i>2∗N/3 **then**30:           Calculate the values of $F3_{-}d$ and *V* according to Equations (15) and (4);31:           Update the particle according to Equation (16);32:           Calculate the fitness value of the particle;33:        **end if**34:    **end for**35:    **for** i=1:N **do**36:        **if** The historical optimal fitness value of the particle < Updated particle optimal fitness value **then**37:           Replace the updated particle position and fitness value with the particles’ optimal historical position and fitness value;38:        **end if**39:    **end for**40:    Backup of the particle’s historical optimal position and its fitness value;41:    Update optimal global position and optimal global value;42:    iter=iter+1;43:**end while**

### 2.2. Mobile Sensor Localization Problem

This section introduces a localization method called Monte Carlo Localization (MCL) for mobile sensor networks, as described in references [39,40]. In wireless sensor networks, Monte Carlo localization methods typically involve fixed anchor nodes. These anchor nodes serve as reference points in the localization algorithm, and their positions are known in advance and remain unchanged over time. During the localization process, anchor nodes send signals to the mobile node and receive signals back from it, aiding in determining the mobile node’s position.

The Monte Carlo localization method is a probabilistic and statistical-based algorithm used to estimate the location of a mobile node through multiple random simulations. It calculates the position of the mobile node using measurements such as received signal strength, arrival time, or other relevant data. The algorithm relies on important parameters, among which the pre-known position of the anchor node plays a crucial role.

In Monte Carlo localization methods, the use of multiple fixed anchor nodes enables the provision of additional measurements for estimating the position of the mobile node. This, in turn, improves the accuracy of the localization process. The fixed positions of the anchor nodes, along with reliable measurement data, form the foundation for the effectiveness of the Monte Carlo localization method in achieving accurate localization.

**Figure 1:**
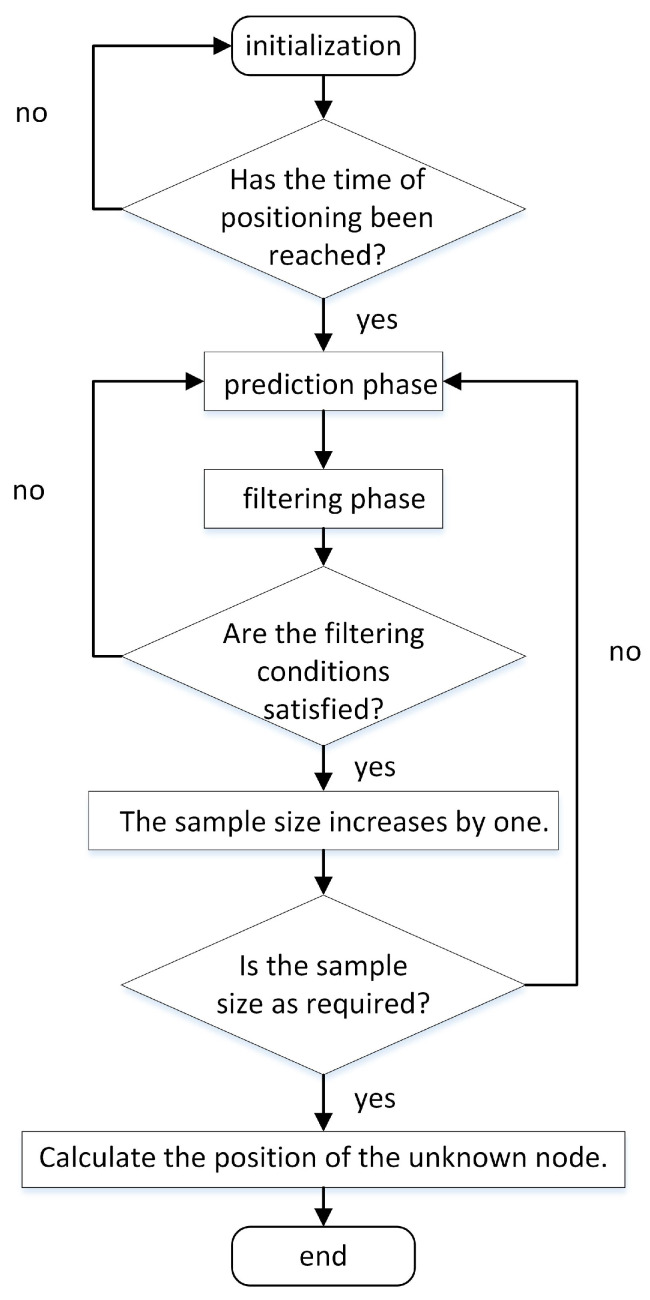
Flowchart of the MCL algorithm.

The MCL (Monte Carlo Localization) method consists of three main phases: initialization, prediction, and filtering [41]. In the initialization phase, each node is assigned motion regions and maximum motion speeds. During the prediction phase, a preliminary estimate of the mobile node’s location is calculated. This estimate corresponds to a circular region, where the last known position of the node serves as the center, and the product of the velocity and positioning interval time determines the radius. Figure 1 illustrates the execution flow of the MCL algorithm.

The filtering phase plays a crucial role in MCL. Initially, MCL calculates the set of single-hop beacon nodes, denoted as S1, and the set of two-hop beacon nodes, denoted as S2, based on their distances to other nodes. Subsequently, MCL randomly selects points within the feasible region and checks if they belong to the set of unknown nodes by verifying if they fall within the range of either single-hop or two-hop beacon nodes. Specifically, a selected point is classified as an unknown node if its nearest anchor is within the range of S1, or if both its closest and next closest anchors fall within the range of S2. Points that fail to satisfy these criteria are filtered out. The filtering condition is expressed in Equation (20).

(20) $$\begin{array}{cc} filer(node) & =\left(\forall s_{1}\in S1,distence\left(node,s_{1}\right)\leq R\right)\cup \left(\forall s_{2}\in S2,R\leq distence\left(node,s_{2}\right)\leq 2R\right) \end{array}$$

**Figure 2:**
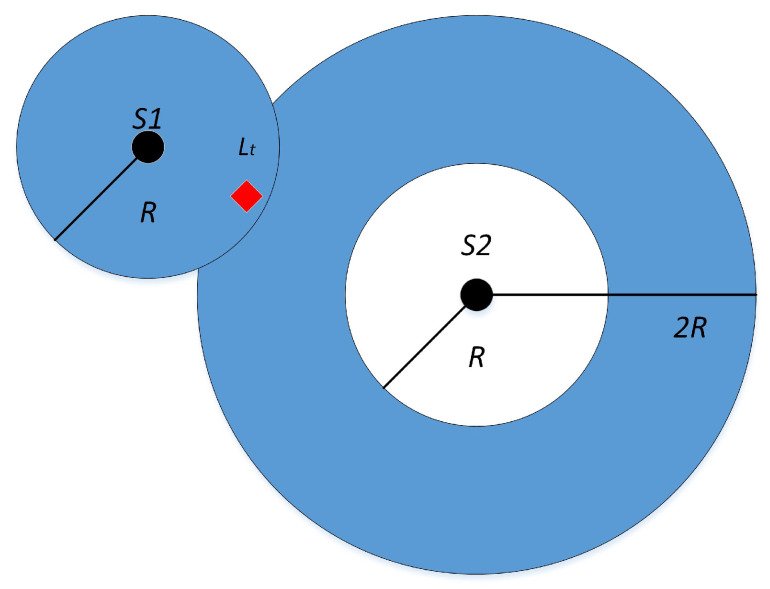
Filtering stage.

As shown in Figure 2, the unknown node *L* senses the information of the surrounding anchor nodes at the moment *t*, where S1 is its one-hop anchor node and S2 is its two-hop anchor node, and the estimated coordinate sample of the unknown node *L* is a valid sample only if it satisfies the filter condition that the distance from S1 is less than *R* and the distance from S2 is between *R* and 2R, Lt in the figure meets the filter condition, and is retained as a reasonable sample particle.

After the filtering phase, numerous sample particles’ coordinates are eliminated, resulting in an insufficient number of sample sets. Hence, the prediction and filtering phases are iteratively executed until an adequately high number of samples remain in the sample set. Eventually, the arithmetic mean of the sample coordinates is calculated, serving as an estimation for the final node coordinates, thereby concluding the localization at the current moment. Equation (21) was employed to estimate the locations of the unknown nodes based on the filtered reference points.

(21) $$Position\left(s\right)=\left(\sum_{i=1}^{N}node_{i}\right)/N$$

## 3. Enhanced Food Digestion Algorithm

This section introduces three intergroup communication strategies and proposes a concise approach to enhance the food digestion algorithm.

### 3.1. Design of Parallel Strategies

**Figure 3:**
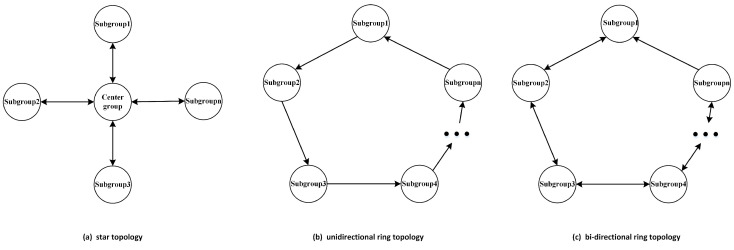
Three topologies.

This section proposes three parallel strategies to speed up the convergence of the algorithm and to improve the algorithm’s optimization finding accuracy. These three parallel strategies use different topologies. Their topologies are shown in Figure 3.

The first parallelization strategy uses a star topology. First, we choose one group as the central group and the others as subgroups. Particles in the central group exchange information with particles in the subgroups, and there is no communication between subgroups. The pseudo-code for the algorithm is shown in Algorithm 2.

The second parallel strategy uses a unidirectional ring topology. The structure allows only subgroups to communicate with their neighboring side, and the side that each group chooses to communicate with is in the same direction in the ring structure. Algorithm 3 shows the details of the communication strategy.
**Algorithm 2** Parallel strategy for star topology1:Calculate the average position of the first three groups of optimal particles and their fitness values;2:**if** The fitness value of the average position < The fitness value of the optimal particle in the central group **then**3:    Replace the position of the central group of optimal particles and its fitness value with the average position and its fitness value;4:**end if**5:Perturbing the central group of optimal particles and calculating its fitness value;6:**if** Particle fitness values after perturbation < The fitness value of the first group of optimal particles **then**7:    Replace the position of the first group of optimal particles and its fitness value with the position of the perturbed particle and its fitness value8:**end if**9:**if** Particle fitness values after perturbation < The fitness value of the second group of optimal particles **then**10:    Replace the position of the second group of optimal particles and its fitness value with the position of the perturbed particle and its fitness value11:**end if**12:**if** Particle fitness values after perturbation < The fitness value of the third group of optimal particles **then**13:    Replace the position of the third group of optimal particles and its fitness value with the position of the perturbed particle and its fitness value14:**end if**

**Algorithm 3** Parallel strategy for unidirectional ring topology
1:**for** g=1:4 **do**2:    Use g+1 to find the remainder of 4 and record the remainder as sg3:    **if** sg==0 **then**4:        sg=45:    **end if**6:    **if** The fitness value of the optimal particle in group *g* > The fitness value of the optimal particle in group sg **then**7:        Replace the optimal particle position and its fitness value of group *g* with the optimal particle position and its fitness value of group sg8:    **else**9:        Disturb the optimal particle of group *g* and calculate its fitness value10:    **end if**11:    **if** Particle fitness value after perturbed < The optimal particle fitness value of group *g* **then**12:        Use the perturbed particle position and its fitness value to replace the optimal particle position and its fitness value of group *g*13:    **end if**14:
**end for**



The third parallel strategy uses a bi-directional ring topology. The structure allows subgroups to exchange information with their neighboring groups, and in a ring structure, subgroups exchange information in a specific direction. Implementation details are given in Algorithm 4.
**Algorithm 4** Parallel strategy for bi-directional ring topology1:**for** g=1:4 **do**2:    Use g+1 to find the remainder of 4 and record the remainder as sg3:    Use g−1 to find the remainder of 4 and record the remainder as vg4:    **if** sg==0 **then**5:        sg=46:    **end if**7:    **if** vg==0 **then**8:        vg=49:    **end if**10:    Calculate the average position of the optimal particle in group sg and group vg and its fitness value11:    **if** The fitness value of the average position < The fitness value of the optimal particle in group *g* **then**12:        Replace the position of the optimal particle in group *g* and its fitness value using the average position and its fitness value13:    **end if**14:**end for**

### 3.2. Design of Compact Strategy

This section describes the principles of the compact mechanism and the detailed process for improving the food digestion algorithm using the compact mechanism.

#### 3.2.1. Principles of the Compact Mechanism

The Distribution Estimation Algorithm (EDA) is a method based on probabilistic models [42]. It maps the population into a probability model and realizes the operation of the population by operating the probability model [43]. Compact algorithms are a type of EDA. It dramatically reduces the use of memory space and speeds up the algorithm’s operation by using a probabilistic model to characterize the distribution of the entire population. The compact algorithm uses a virtual population instead of the actual population. This virtual population is encoded in a PV vector. It is an *N* × 2 matrix in compact differential evolution (CDE) [43] and real-valued compact genetic algorithms (RCGAs) [44].

(22) $$PV=\left[\mu^{t},\delta^{t}\right]$$

μ and δ denote the mean and standard deviation of the PV, respectively, and *t* denotes the current number of iterations. Each pair of mean and standard deviation in PV corresponds to the corresponding Probability Density Function (PDF), which is truncated at [−1, 1] and normalizes the amplitude area to 1 [45]. The calculation of PDF is given by Equation (23).

(23) $$PDF\left(turncNorm\left(x\right)\right)=\frac{\sqrt{\frac{2}{\pi }}e^{-\frac{{\left(x-\mu_{i}\right)}^{2}}{2\delta_{i}^{2}}}}{\delta_{i}\left(erf\left(\frac{\mu_{i}+1}{\sqrt{2}\delta_{i}}\right)-erf\left(\frac{\mu_{i}-1}{\sqrt{2}\delta_{i}}\right)\right)}$$

erf is the error function. By constructing Chebyshev polynomials, PDF can correspond to a Cumulative Distribution Function (CDF) with values ranging from 0 to 1 [46,47]. CDF is calculated as shown in Equation (24):

(24) $$CDF=\int_{-1}^{x}PDF\;dx=\int_{-1}^{x}\frac{\sqrt{\frac{2}{\pi }}e^{-\frac{{\left(x-\mu_{i}\right)}^{2}}{2\delta_{i}^{2}}}}{\delta_{i}\left(erf\left(\frac{\mu_{i}+1}{\sqrt{2}\delta_{i}}\right)-erf\left(\frac{\mu_{i}-1}{\sqrt{2}\delta_{i}}\right)\right)}dx$$

In Equation (24), *x* takes values in the range [−1, 1]. The function CDF can be expressed as Equation (25):

(25) $$CDF=\frac{erf\left(\frac{\mu +1}{\sqrt{2}\delta }\right)+erf\left(\frac{x-\mu }{\sqrt{2}\delta }\right)}{erf\left(\frac{\mu +1}{\sqrt{2}\delta }\right)-erf\left(\frac{\mu -1}{\sqrt{2}\delta }\right)}$$

CDF returns the value in the range [0, 1].

The process of sampling the design variable $X_{i}$ from the PV vector is to first generate a random number *R* from a uniform distribution and then calculate its corresponding inverse function of CDF to obtain a new value. This newly generated value is compared with another value, with the one with the better fitness value being the winner and the one with the worse fitness value being the loser, both of which are retained for updating the PV vector. The updated equations of mean and standard deviation are shown in Equations (26) and (27).

(26) $$\mu_{i}^{t+1}=\mu_{i}^{t}+\frac{1}{N}\left({winner}_{i}-{loser}_{i}\right)$$

(27) $$\delta_{i}^{t+1}=\sqrt{{\left(\delta_{i}^{t}\right)}^{2}+{\left(\mu_{i}^{t}\right)}^{2}-{\left(\mu_{i}^{t+1}\right)}^{2}+\frac{1}{N_{p}}\left({winner}_{i}^{2}-{loser}_{i}^{2}\right)}$$

$N_{p}$ denotes the size of the virtual population, which is a typical parameter of compact algorithms, and the size of this parameter is usually several times the size of the actual population [44].

#### 3.2.2. Compact Food Digestion Algorithm

Compact algorithms reduce memory space usage and speed up algorithms, but they reduce population diversity and tend to fall into local optima. A solution is generated by sampling from the probabilistic model during each iteration to solve this problem. Then three solutions are generated using the sampled solutions in conjunction with the characteristics of the FDA algorithm. These three solutions are generated using the particle update formulae in the oral cavity, stomach, and small intestine. Since the extent of the sampling space is not the same as the actual space, it is essential to map the generated solution $Food_{1}^{t}$ to the actual computational space once it has been sampled in the probabilistic model, and we use Equation (28) to complete this process.

(28) $${Food}_{1}^{t}=\frac{{Food}_{1}^{t}*\left(ub-lb\right)}{2}-\frac{ub+lb}{2}$$

ub and lb are the maximum and minimum bounds on the actual space, respectively. The updated equation for the three solutions is given by Equations (29)–(31).

(29) $$\begin{array}{cc} {Food}_{2}^{t}= & {Food}_{1}^{t}+F1_{-}d*\left(C1*{Best}_{-}p-{Food}_{1}^{t}\right)+C1*Em*\left({Food}_{1}^{t}-C2*group\left(g\right).{Best}_{-}p\right)*V \end{array}$$

(30) $$\begin{array}{cc} {Food}_{3}^{t}= & {Food}_{2}^{t}+F2_{-}d*\left({Food}_{1}^{t}-C1*{Food}_{2}^{t}\right)+C1*Em*\left({Food}_{2}^{t}-Mean\right)*V \end{array}$$

(31) $$\begin{array}{cc} {Food}_{4}^{t}= & {Best}_{-}p+F3_{-}d*\left({Food}_{2}^{t}-C1*{Food}_{3}^{t}\right)+C1*Em*Levy\left(D\right)*V \end{array}$$

$Food_{2}^{t}$ is the particle generated using the particle update equation in the oral cavity, where $Food_{1}^{t}$ is the particle generated by sampling from the probabilistic model, $Best_{-}p$ is the optimal global particle, and $group\left(g\right).Best_{-}p$ is the optimal particle of the gth group. $Food_{3}^{t}$ is the particle generated using the particle update equation in the stomach, and Mean is the particle obtained by averaging $Food_{1}^{t}$ and $Food_{2}^{t}$. $Food_{4}^{t}$ is the particle generated using the particle update equation in the small intestine. The meaning of the other variables in these three equations is the same as in the FDA in Section 2. The pseudo-code of the FDA algorithm for the parallel compact strategy is shown in Algorithm 5.
**Algorithm 5** Parallel Compact Food Digestion Algorithm**Output:** Population size $N_{p}$; Dimension *D*; Maximum number of iterations $Max_{-}iter$; Lower boundary lb; Upper boundary ub;**Input:** Global optimal position $Best_{-}p$, Global optimal fitness value $Best_{-}f$;1:Initialize the parameters $a,b,a_{1},K_{m},V_{max},iter$ and the number of groups groups as well as the mean and standard deviation μ and δ for each group;2:**while** $iter<Max_{-}iter$ **do**3:    **for** i=1:groups **do**4:        Sampling generates particles $Food_{1}^{t}$ and calculates their fitness values;5:        Calculate the values of $F1,F2,F3,C1,C2,E_{m}$ and *S*;6:        Calculate the values of $F1_{-}d$ and *V*;7:        Update the particle to get $Food_{2}^{t}$ and calculate its fitness value;8:        Calculate the values of $F2_{-}d$ and *V*;9:        Update the particle to get $Food_{3}^{t}$ and calculate its fitness value;10:      Calculate the values of $F3_{-}d$ and *V*;11:      Update the particle to get $Food_{4}^{t}$ and calculate its fitness value;12:      Find the particle with the best and worst fitness value among the four particles, denoted as winner and loser;13:      Use winner and loser to update PV;14:    **end for**15:    Intergroup communication using parallel strategies;16:    Find the global optimal solution $Best_{-}p$ and its fitness value $Best_{-}f$;17:    iter=iter+1;18:**end while**

## 4. Numerical Experimental Results and Analysis

This section not only compares the PCFDA with the original FDA but also compares it with the PCSCA [28]. In reference [28], the authors propose three strategies for parallel communication, which apply to solving single-peak, multi-peak, and mixed-function problems. This section verifies the effectiveness of PCFDA by comparing it with them.

### 4.1. Parameter Settings

In this section, experiments are conducted using a Lenovo computer manufactured in Shanghai, China, equipped with an Intel(R) Core(TM) i3-8100 CPU at 3.60 GHz, 24 GB of RAM, a 64-bit Windows 10 operating system, and MATLAB2018a.

This section uses the CEC2013 test set for test experiments. The test set consists of 28 test functions, including five unimodal, fifteen multimodal, and eight mixed functions. Unimodal functions have only one global optimal solution and are used to test the ability of the algorithm to develop. Multimodal functions have multiple local optimal solutions and are mainly used to test the ability of the algorithm to escape from local optimal solutions. Mixed functions are extremely complex, they have the characteristics of both single-peak and multi-peak functions, and can test both the development ability of the algorithm and the ability of the algorithm to escape from the local optimal solution, which is the function that can best reflect the ability of the algorithm to solve complex problems. Using these three types of function tests to test the metaheuristic algorithm can effectively assess the performance and reliability of the algorithm and improve the practical application value of the algorithm.

To ensure the experiments’ fairness and reduce the effect of algorithmic instability, we let all algorithms run ten times on 28 test functions for 1000 iterations. Finally, the mean and standard deviation of their runs on each function are compared. The dimension of each particle is set to 30, and the range of the particle search is in the range [−100, 100]. The number of groups in the algorithm is set to 4, and the initial mean and standard deviation values are set to 0 and 10. The number of particles in the FDA is set to 20. $K_{m}$ has three different values that indicate the three characteristic constant values of the algorithm in the oral cavity, stomach, and small intestine, which have values of 0.8, 0.9, and 1, respectively. The parameter settings of PCSCA follow the original paper, and its three algorithms are denoted by PCSCAS1, PCSCAS2, and PCSCAS3, respectively. For the experiments in this section, we use PCFDA1, PCFDA2, and PCFDA3 to represent the enhanced FDA using Algorithms 2–4.

### 4.2. Comparison with the Original FDA

In this section, we use PCFDA to compare with the original FDA, mainly comparing the mean and standard deviation of their runs on each function as well as the time cost and memory usage of their runs to determine the performance of PCFDA. The mean and standard deviation comparison results are shown in Table 1 and Table 2.

In Table 1 and Table 2, the data in the last row indicate the number of PCFDAs better than the FDA. On the Unimodal functions f1–f5, PCFDA1 has a better searching ability on the first three functions and is more stable on f2 and f3. On the Multimodal functions f6–f20, all the algorithms have good searchability and stability on f8 and f20. PCFDA3’s search ability is poor on Multimodal functions. FDA and PCFDA2, and PCFDA3 outperformed on different Multimodal functions with comparable performance. On the Mixed functions f21–f28, PCFDA2 has better searchability and stability on four functions, while PCFDA3 only performs better on f26. Overall, PCFDA1 and PCFDA2 are comparable to the original FDA regarding merit-seeking ability but are more stable than the FDA. PCFDA3 has improved performance on a few functions, but overall performance is not as good as the FDA.

In order to statistically verify the effectiveness of the improved algorithm, this paper uses the Wilkerson rank sum test to verify the significant difference between the improved algorithm and the original algorithm. The significance level alpha is set to 0.05. Table 3 displays the *p*-values for the comparison results. The data with *p*-values less than 0.05 are highlighted in red. From the data in the table, it can be observed that the improved algorithm holds a significant advantage.

Improving the algorithms using compact strategies is more concerned with the time cost and the memory footprint size. Table 4 shows the time loss and memory usage for each algorithm.

In Table 4, the average running time indicates the average time to run each algorithm 10 times on 28 functions, the memory usage indicates the memory space occupied by each particle in each algorithm, the * is used as a multiplication sign, and *D* indicates the particle dimension. (20+1)∗D denotes the memory occupied by the 20 particles in the FDA and one globally optimal particle. In the last three columns of Table 4, (2∗4)∗D denotes the memory occupied by μ and δ in the four groups. The following two 4s represent the memory occupied by the four particles obtained from each update (including one sampled particle and three generated particles) and the optimal particle in the four groups, respectively. The last 1 denotes the memory occupied by a temporary particle needed in the communication strategy. Combining the results of each algorithm in Table 1 and Table 2 leads to the conclusion that the improved algorithms are improved in terms of both time cost and memory space.

### 4.3. Comparison with PCSCA

This section compares the improved FDA with PCSCA. Both algorithms use parallel and compact strategies for improvement, so we only compare their searchability and stability here. Table 5 and Table 6 show the mean and standard deviation comparison results.

The red font in Table 5 and Table 6 indicates the mean and standard deviation of the optimum found by each algorithm on each function. As seen from the tables, on the f20, all algorithms show good searching ability and stability. On the f8, all algorithms have the same search ability, but PCSCAS3 is more stable. On the other functions, the PCFDA outperformed the PCSCA regarding searching superiority.

In this section, the Wilcoxon rank sum test was also used for the significance analysis of the proposed algorithm in this paper. We conducted significance analysis of the three algorithms proposed in this paper with the parallel compact SCA algorithm. Table 7, Table 8 and Table 9 display the comparison results, with red font indicating data with *p*-values greater than 0.05. From the data in the table, it can be observed that the proposed algorithm in this paper outperforms the parallel compact SCA algorithm in most functions.

### 4.4. Convergence Analysis

**Figure 4:**
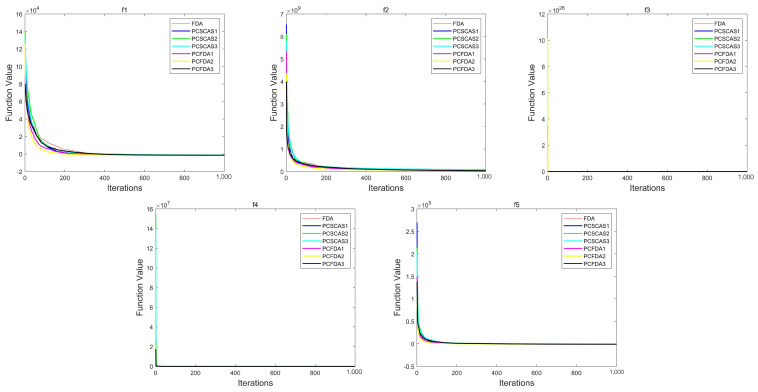
Convergence curves on the unimodal functions.

**Figure 5:**
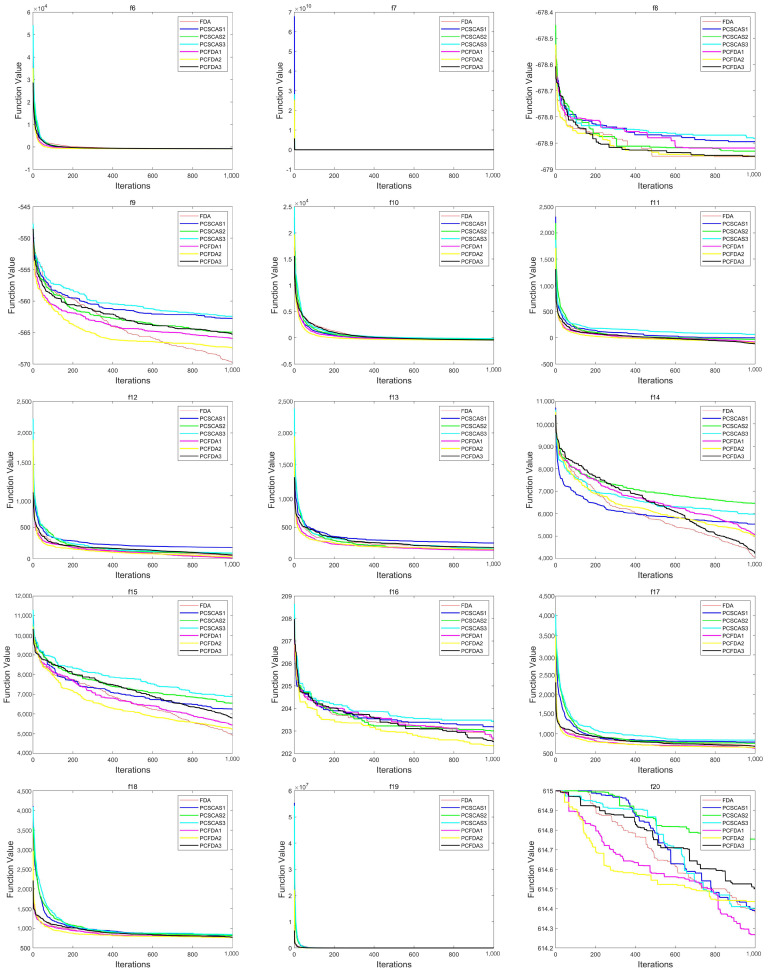
Convergence curves on multimodal functions.

**Figure 6:**
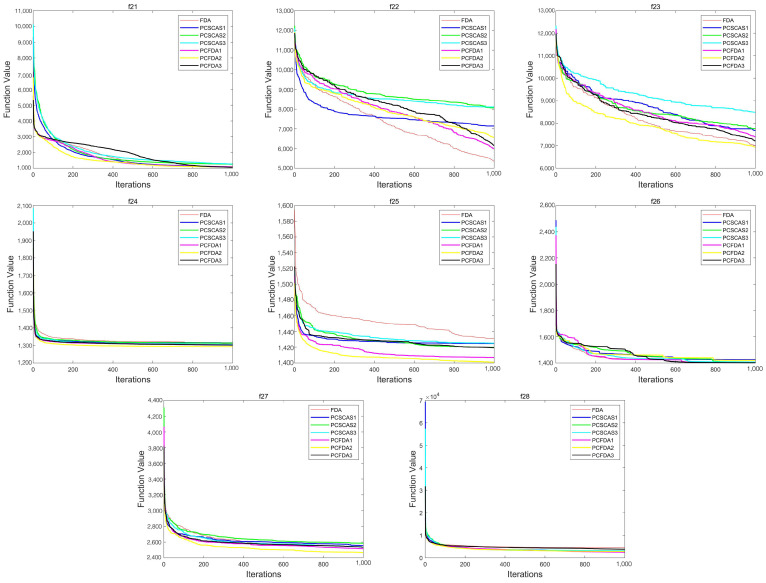
Convergence curves on mixed functions.

This section evaluates the performance of the algorithms by comparing the convergence curves of the PCFDA and PCSCA algorithms on three classes of functions. Figure 4, Figure 5 and Figure 6 show the corresponding experimental results.

From the convergence curves of the three types of functions, on the unimodal function, the convergence speed of each algorithm is not much different. Only on f1 do the PCFDA1 and PCFDA2 algorithms converge faster in the early stage. On the multimodal functions f8 and f20, although the convergence speeds of the algorithms are quite different, they have similar optimization capabilities based on the data in Table 1 and Table 5. On f6, f7, f10, and f19, the convergence speed of each algorithm is similar. Due to the instability of each algorithm’s search on other multimodal functions, the convergence speed and accuracy are different. On the mixed functions f23, f24, f25, and f27, PCFDA2 converges faster and has the best optimization accuracy. On the function f22, the FDA has better convergence speed and accuracy than other algorithms.

## 5. Application of PCFDA in Mobile Sensor Localization Problem

This section discusses the PCFDA algorithm for mobile sensor localization and compares it with the original MCL algorithm under different numbers of anchor nodes and communication radii. Locations with large errors are first obtained by the MCL localization technique, and then the PCFDA algorithm is applied for further optimization around the obtained locations to reduce the localization error. The error function is defined as Equation (32):

(32) $$error=\frac{\sum_{k=1}^{Z}\left(\sum_{l=1,l\neq k}^{N}\sqrt{{\left(x_{l}-x_{k}\right)}^{2}+{\left(y_{l}-y_{k}\right)}^{2}}-D_{lk}\right)}{Z}$$

*Z* represents the total number of unknown nodes, and *N* represents the total number of anchor nodes. $\left(x_{l},y_{k}\right)$ denotes the estimated location of the unknown node *l*, and $\left(x_{k},y_{k}\right)$ denotes the location of the anchor node. $D_{lk}$ represents the distance between unknown node *l* and anchor node *k*. This section assumes that anchor node *k* can obtain the distance between anchor node *k* and unknown node *l* through the signal strength received from unknown node *l*. The smaller the error value, the higher the positioning accuracy.

### 5.1. Experimental Analysis of Different Numbers of Anchor Nodes

In this section, the total number of nodes is set to 300, randomly distributed within the space range of 300 × 300. The number of anchor nodes is set to 10, 20, 30, 40, and 50, and the communication radius is set to 50. Experiments were performed using the MCL localization algorithm, FDA, and PCFDA. To avoid randomness, this section runs each algorithm 10 times and takes the average of 10 runs as the final result. The experimental results are shown in Table 10.

In Table 10, Ave and Std represent the mean and standard deviation of the run results, respectively. It can be seen from Table 10 that under the condition of a fixed communication radius, the more the number of anchor nodes, the smaller the positioning error and the more accurate the positioning. Compared with the MCL positioning algorithm, the positioning accuracy of the FDA has improved a lot, but the FDA is extremely unstable. The cAPSO [48] algorithm has comparable localization accuracy to the FDA algorithm, but it is more stable than the FDA algorithm. Under the same experimental conditions, the performance of the PCFDA is remarkable, both in positioning accuracy and algorithm stability are better than the FDA, and the positioning accuracy is much better than the MCL algorithm.

### 5.2. Experimental Analysis of Different Communication Radius

This section also uses 300 nodes for experiments and distributes them in the space of 300 × 300. The number of anchor nodes is set to 50, and the communication radius is set to 20, 40, 60, and 80, respectively. Each algorithm is run 10 times in this section, and the mean and standard deviation of 10 runs are taken for experimental analysis. The experimental results are shown in Table 11.

Table 11 shows that when the number of anchor nodes is fixed, the larger the communication radius, the smaller the positioning error, and the more accurate the positioning. The positioning accuracy of the FDA is better than the MCL positioning algorithm, but the stability is poor. The cAPSO algorithm is comparable to the FDA algorithm in terms of localization accuracy, but with better stability. The performance improvement of PCFDA is more significant and has good results in positioning accuracy and operational stability.

## 6. Conclusions

This paper proposes three intergroup communication strategies to improve the food digestion algorithm. These three strategies use different topologies, which significantly demonstrate the efficiency of particle communication and speed up the algorithm’s convergence. This paper also uses a compact strategy to improve the food digestion algorithm, reducing the algorithm’s running time and saving memory space. Then, this paper tested the PCFDA algorithm on the CEC2013 test set and achieved good results. Finally, this paper uses the improved algorithm to solve the problem of mobile sensor localization, which reduces the error of positioning and improves the accuracy of positioning.

In the future, we can use other inter-group communication strategies to further improve the FDA’s search accuracy. In the meantime, we will consider using the improved algorithm for other localization problems in wireless sensor networks. The design process of the algorithm does not take into account issues such as communication barriers of mobile sensors in real environments, so these factors can be considered to be added in future research.

## Figures and Tables

**Table 1:** The average of the running results of the improved FDA and the original FDA.

Functions	FDA	PCFDA1	PCFDA2	PCFDA3
f1	−$4.61\times 10^{2}$	−$4.63\times 10^{2}$	−$4.63\times 10^{2}$	−$4.63\times 10^{2}$
f2	$1.30\times 10^{7}$	$9.94\times 10^{6}$	$1.35\times 10^{7}$	$1.24\times 10^{7}$
f3	$4.51\times 10^{9}$	$3.02\times 10^{9}$	$3.17\times 10^{9}$	$3.35\times 10^{9}$
f4	$1.43\times 10^{4}$	$1.66\times 10^{4}$	$1.61\times 10^{4}$	$1.75\times 10^{4}$
f5	−$3.17\times 10^{2}$	−$3.07\times 10^{2}$	−$3.08\times 10^{2}$	−$3.01\times 10^{2}$
f6	−$2.61\times 10^{2}$	−$2.58\times 10^{2}$	−$2.73\times 10^{2}$	−$2.58\times 10^{2}$
f7	−$2.13\times 10^{2}$	−$2.13\times 10^{2}$	−$2.02\times 10^{2}$	−$2.15\times 10^{2}$
f8	−$2.26\times 10^{2}$	−$2.26\times 10^{2}$	−$2.26\times 10^{2}$	−$2.26\times 10^{2}$
f9	−$1.90\times 10^{2}$	−$1.89\times 10^{2}$	−$1.89\times 10^{2}$	−$1.88\times 10^{2}$
f10	−$1.50\times 10^{2}$	−$1.49\times 10^{2}$	−$1.56\times 10^{2}$	−$1.46\times 10^{2}$
f11	−$3.99\times 10^{1}$	−$2.70\times 10^{1}$	−$3.49\times 10^{1}$	−$3.82\times 10^{1}$
f12	$1.58\times 10^{1}$	$3.38\times 10^{0}$	$9.36\times 10^{0}$	$1.95\times 10^{1}$
f13	$5.86\times 10^{1}$	$4.52\times 10^{1}$	$5.02\times 10^{1}$	$5.95\times 10^{1}$
f14	$1.35\times 10^{3}$	$1.69\times 10^{3}$	$1.66\times 10^{3}$	$1.41\times 10^{3}$
f15	$1.63\times 10^{3}$	$1.81\times 10^{3}$	$1.75\times 10^{3}$	$1.93\times 10^{3}$
f16	$6.75\times 10^{1}$	$6.76\times 10^{1}$	$6.74\times 10^{1}$	$6.75\times 10^{1}$
f17	$2.30\times 10^{2}$	$2.09\times 10^{2}$	$2.15\times 10^{2}$	$2.26\times 10^{2}$
f18	$2.59\times 10^{2}$	$2.56\times 10^{2}$	$2.55\times 10^{2}$	$2.56\times 10^{2}$
f19	$1.77\times 10^{2}$	$1.76\times 10^{2}$	$1.75\times 10^{2}$	$1.76\times 10^{2}$
f20	$2.05\times 10^{2}$	$2.05\times 10^{2}$	$2.05\times 10^{2}$	$2.05\times 10^{2}$
f21	$3.60\times 10^{2}$	$3.63\times 10^{2}$	$3.55\times 10^{2}$	$3.51\times 10^{2}$
f22	$1.78\times 10^{3}$	$2.00\times 10^{3}$	$2.19\times 10^{3}$	$2.05\times 10^{3}$
f23	$2.33\times 10^{3}$	$2.46\times 10^{3}$	$2.31\times 10^{3}$	$2.40\times 10^{3}$
f24	$4.36\times 10^{2}$	$4.34\times 10^{2}$	$4.31\times 10^{2}$	$4.34\times 10^{2}$
f25	$4.77\times 10^{2}$	$4.69\times 10^{2}$	$4.67\times 10^{2}$	$4.73\times 10^{2}$
f26	$4.67\times 10^{2}$	$4.67\times 10^{2}$	$4.73\times 10^{2}$	$4.67\times 10^{2}$
f27	$8.44\times 10^{2}$	$8.38\times 10^{2}$	$8.22\times 10^{2}$	$8.45\times 10^{2}$
f28	$1.51\times 10^{3}$	$8.20\times 10^{2}$	$9.33\times 10^{2}$	$1.26\times 10^{3}$
win		14	16	12

**Table 2:** The standard deviation of the running results of the improved FDA and the original FDA.

Functions	FDA	PCFDA1	PCFDA2	PCFDA3
f1	$6.63\times 10^{2}$	$6.67\times 10^{2}$	$6.65\times 10^{2}$	$6.67\times 10^{2}$
f2	$1.98\times 10^{7}$	$1.57\times 10^{7}$	$2.19\times 10^{7}$	$2.04\times 10^{7}$
f3	$7.67\times 10^{9}$	$5.02\times 10^{9}$	$5.14\times 10^{9}$	$6.21\times 10^{9}$
f4	$2.07\times 10^{4}$	$2.46\times 10^{4}$	$2.38\times 10^{4}$	$2.53\times 10^{4}$
f5	$4.56\times 10^{2}$	$4.42\times 10^{2}$	$4.43\times 10^{2}$	$4.33\times 10^{2}$
f6	$3.76\times 10^{2}$	$3.73\times 10^{2}$	$3.94\times 10^{2}$	$3.72\times 10^{2}$
f7	$3.07\times 10^{2}$	$3.07\times 10^{2}$	$2.92\times 10^{2}$	$3.10\times 10^{2}$
f8	$3.26\times 10^{2}$	$3.26\times 10^{2}$	$3.26\times 10^{2}$	$3.26\times 10^{2}$
f9	$2.73\times 10^{2}$	$2.71\times 10^{2}$	$2.72\times 10^{2}$	$2.71\times 10^{2}$
f10	$2.16\times 10^{2}$	$2.15\times 10^{2}$	$2.25\times 10^{2}$	$2.10\times 10^{2}$
f11	$7.51\times 10^{1}$	$4.99\times 10^{1}$	$6.29\times 10^{1}$	$7.90\times 10^{1}$
f12	$4.34\times 10^{1}$	$4.03\times 10^{1}$	$2.85\times 10^{1}$	$5.50\times 10^{1}$
f13	$9.32\times 10^{1}$	$7.37\times 10^{1}$	$7.81\times 10^{1}$	$9.82\times 10^{1}$
f14	$2.00\times 10^{3}$	$2.51\times 10^{3}$	$2.47\times 10^{3}$	$2.08\times 10^{3}$
f15	$2.36\times 10^{3}$	$2.66\times 10^{3}$	$2.56\times 10^{3}$	$2.81\times 10^{3}$
f16	$9.71\times 10^{1}$	$9.72\times 10^{1}$	$9.70\times 10^{1}$	$9.71\times 10^{1}$
f17	$3.33\times 10^{2}$	$3.02\times 10^{2}$	$3.10\times 10^{2}$	$3.27\times 10^{2}$
F18	$3.74\times 10^{2}$	$3.70\times 10^{2}$	$3.68\times 10^{2}$	$3.70\times 10^{2}$
f19	$2.55\times 10^{2}$	$2.53\times 10^{2}$	$2.51\times 10^{2}$	$2.53\times 10^{2}$
f20	$2.95\times 10^{2}$	$2.95\times 10^{2}$	$2.95\times 10^{2}$	$2.95\times 10^{2}$
f21	$5.19\times 10^{2}$	$5.23\times 10^{2}$	$5.13\times 10^{2}$	$5.07\times 10^{2}$
f22	$2.59\times 10^{3}$	$2.91\times 10^{3}$	$3.19\times 10^{3}$	$3.00\times 10^{3}$
f23	$3.37\times 10^{3}$	$3.60\times 10^{3}$	$3.34\times 10^{3}$	$3.48\times 10^{3}$
f24	$6.28\times 10^{2}$	$6.25\times 10^{2}$	$6.20\times 10^{2}$	$6.24\times 10^{2}$
f25	$6.86\times 10^{2}$	$6.75\times 10^{2}$	$6.72\times 10^{2}$	$6.80\times 10^{2}$
f26	$6.72\times 10^{2}$	$6.72\times 10^{2}$	$6.81\times 10^{2}$	$6.72\times 10^{2}$
f27	$1.21\times 10^{3}$	$1.21\times 10^{3}$	$1.18\times 10^{3}$	$1.22\times 10^{3}$
f28	$2.19\times 10^{3}$	$1.46\times 10^{3}$	$1.55\times 10^{3}$	$1.97\times 10^{3}$
win		18	18	14

**Table 3:** The comparison results between PCFDA1, PCFDA2, and PCFDA3 with FDA.

Functions	PCFDA1	PCFDA2	PCFDA3
f1	$4.396\times 10^{-4}$	$1.706\times 10^{-3}$	$1.827\times 10^{-4}$
f2	$2.123\times 10^{-2}$	$3.847\times 10^{-1}$	$3.728\times 10^{-2}$
f3	$4.708\times 10^{-2}$	$4.897\times 10^{-2}$	$2.113\times 10^{-2}$
f4	$5.133\times 10^{-2}$	$5.828\times 10^{-2}$	$5.827\times 10^{-2}$
f5	$5.827\times 10^{-2}$	$5.827\times 10^{-2}$	$5.827\times 10^{-1}$
f6	$4.727\times 10^{-1}$	$3.447\times 10^{-2}$	$4.727\times 10^{-1}$
f7	$9.108\times 10^{-3}$	$5.575\times 10^{-2}$	$9.108\times 10^{-3}$
f8	$2.413\times 10^{-2}$	$3.764\times 10^{-2}$	$4.708\times 10^{-2}$
f9	$5.586\times 10^{-2}$	$4.396\times 10^{-4}$	$5.827\times 10^{-2}$
f10	$2.413\times 10^{-1}$	$4.396\times 10^{-4}$	$5.827\times 10^{-2}$
f11	$2.730\times 10^{-1}$	$5.019\times 10^{-2}$	$5.764\times 10^{-2}$
f12	$7.337\times 10^{-3}$	$1.859\times 10^{-2}$	$3.075\times 10^{-1}$
f13	$4.727\times 10^{-2}$	$4.097\times 10^{-2}$	$3.847\times 10^{-1}$
f14	$5.133\times 10^{-2}$	$5.402\times 10^{-2}$	$5.396\times 10^{-2}$
f15	$7.337\times 10^{-2}$	$1.133\times 10^{-1}$	$1.726\times 10^{-1}$
f16	$7.913\times 10^{-1}$	$4.708\times 10^{-2}$	$1.859\times 10^{-2}$
f17	$4.501\times 10^{-2}$	$4.097\times 10^{-2}$	$4.910\times 10^{-2}$
f18	$4.274\times 10^{-2}$	$2.123\times 10^{-2}$	$1.620\times 10^{-2}$
f19	$3.708\times 10^{-2}$	$4.274\times 10^{-2}$	$4.897\times 10^{-2}$
f20	$9.108\times 10^{-3}$	$3.890\times 10^{-2}$	$4.274\times 10^{-2}$
f21	$1.212\times 10^{-1}$	$1.133\times 10^{-2}$	$1.405\times 10^{-2}$
f22	$1.212\times 10^{-1}$	$1.405\times 10^{-1}$	$5.586\times 10^{-2}$
f23	$9.097\times 10^{-1}$	$3.447\times 10^{-2}$	$6.776\times 10^{-1}$
f24	$4.586\times 10^{-3}$	$4.791\times 10^{-2}$	$3.075\times 10^{-2}$
f25	$3.298\times 10^{-4}$	$2.123\times 10^{-4}$	$2.461\times 10^{-4}$
f26	$3.764\times 10^{-2}$	$7.685\times 10^{-2}$	$4.396\times 10^{-4}$
f27	$3.678\times 10^{-2}$	$3.910\times 10^{-2}$	$7.913\times 10^{-2}$
f28	$3.764\times 10^{-2}$	$1.620\times 10^{-1}$	$1.008\times 10^{-3}$

**Table 4:** The average running time and memory usage of each algorithm.

Evaluation Indicators	FDA	PCFDA1	PCFDA2	PCFDA3
Average running time	36.34	33.22	30.37	35.46
Memory usage	(20+1)∗D	(2∗4+4+4+1)∗D	(2∗4+4+4+1)∗D	(2∗4+4+4+1)∗D

**Table 5:** The running results of the average value of each algorithm.

Functions	PCSCAS1	PCSCAS2	PCSCAS3	PCFDA1	PCFDA2	PCFDA3
f1	−$4.54\times 10^{2}$	−$3.70\times 10^{2}$	−$4.04\times 10^{2}$	−$4.63\times 10^{2}$	−$4.63\times 10^{2}$	−$4.63\times 10^{2}$
f2	$2.07\times 10^{7}$	$2.43\times 10^{7}$	$3.02\times 10^{7}$	$9.94\times 10^{6}$	$1.35\times 10^{7}$	$1.24\times 10^{7}$
f3	$9.75\times 10^{9}$	$5.49\times 10^{9}$	$9.07\times 10^{9}$	$3.02\times 10^{9}$	$3.17\times 10^{9}$	$3.35\times 10^{9}$
f4	$3.17\times 10^{4}$	$3.49\times 10^{4}$	$3.27\times 10^{4}$	$1.66\times 10^{4}$	$1.61\times 10^{4}$	$1.75\times 10^{4}$
f5	−$2.77\times 10^{2}$	−$1.76\times 10^{2}$	−$1.77\times 10^{2}$	−$3.07\times 10^{2}$	−$3.08\times 10^{2}$	−$3.01\times 10^{2}$
f6	−$2.55\times 10^{2}$	−2.52$\times 10^{2}$	−$2.54\times 10^{2}$	−$2.58\times 10^{2}$	−$2.73\times 10^{2}$	−$2.58\times 10^{2}$
f7	−$1.89\times 10^{2}$	−$1.95\times 10^{2}$	−$1.96\times 10^{2}$	−$2.13\times 10^{2}$	−$2.02\times 10^{2}$	−$2.15\times 10^{2}$
f8	−$2.26\times 10^{2}$	−$2.26\times 10^{2}$	−$2.26\times 10^{2}$	−$2.26\times 10^{2}$	−$2.26\times 10^{2}$	−$2.26\times 10^{2}$
f9	−$1.88\times 10^{2}$	−$1.88\times 10^{2}$	−$1.87\times 10^{2}$	−$1.89\times 10^{2}$	−$1.89\times 10^{2}$	−$1.88\times 10^{2}$
f10	−$1.16\times 10^{2}$	−$9.25\times 10^{1}$	−$6.32\times 10^{1}$	−$1.49\times 10^{2}$	−$1.56\times 10^{2}$	−$1.46\times 10^{2}$
f11	−1.35	−$1.07\times 10^{1}$	$2.13\times 10^{1}$	−$2.70\times 10^{1}$	−$3.49\times 10^{1}$	−$3.82\times 10^{1}$
f12	$5.91\times 10^{1}$	$2.85\times 10^{1}$	$2.96\times 10^{1}$	3.38	9.36	$1.95\times 10^{1}$
f13	$8.30\times 10^{1}$	$5.18\times 10^{1}$	$5.33\times 10^{1}$	$4.52\times 10^{1}$	$5.02\times 10^{1}$	$5.95\times 10^{1}$
f14	$1.85\times 10^{3}$	$2.15\times 10^{3}$	$1.99\times 10^{3}$	$1.69\times 10^{3}$	$1.66\times 10^{3}$	$1.41\times 10^{3}$
f15	$2.08\times 10^{3}$	$2.18\times 10^{3}$	$2.30\times 10^{3}$	$1.81\times 10^{3}$	$1.75\times 10^{3}$	$1.93\times 10^{3}$
f16	$6.77\times 10^{1}$	$6.77\times 10^{1}$	$6.78\times 10^{1}$	$6.76\times 10^{1}$	$6.74\times 10^{1}$	$6.75\times 10^{1}$
f17	$2.63\times 10^{2}$	$2.53\times 10^{2}$	$2.80\times 10^{2}$	$2.09\times 10^{2}$	$2.15\times 10^{2}$	$2.26\times 10^{2}$
f18	$2.72\times 10^{2}$	$2.73\times 10^{2}$	$2.81\times 10^{2}$	$2.56\times 10^{2}$	$2.55\times 10^{2}$	$2.56\times 10^{2}$
f19	$1.82\times 10^{2}$	$1.79\times 10^{2}$	$1.84\times 10^{2}$	$1.76\times 10^{2}$	$1.75\times 10^{2}$	$1.76\times 10^{2}$
f20	$2.05\times 10^{2}$	$2.05\times 10^{2}$	$2.05\times 10^{2}$	$2.05\times 10^{2}$	$2.05\times 10^{2}$	$2.05\times 10^{2}$
f21	$3.66\times 10^{2}$	$4.15\times 10^{2}$	$4.20\times 10^{2}$	$3.63\times 10^{2}$	$3.55\times 10^{2}$	$3.51\times 10^{2}$
f22	$2.38\times 10^{3}$	$2.69\times 10^{3}$	$2.70\times 10^{3}$	$2.00\times 10^{3}$	$2.19\times 10^{3}$	$2.05\times 10^{3}$
f23	$2.56\times 10^{3}$	$2.59\times 10^{3}$	$2.83\times 10^{3}$	$2.46\times 10^{3}$	$2.31\times 10^{3}$	$2.40\times 10^{3}$
f24	$4.38\times 10^{2}$	$4.38\times 10^{2}$	$4.36\times 10^{2}$	$4.34\times 10^{2}$	$4.31\times 10^{2}$	$4.34\times 10^{2}$
f25	$4.75\times 10^{2}$	$4.73\times 10^{2}$	$4.75\times 10^{2}$	$4.69\times 10^{2}$	$4.67\times 10^{2}$	$4.73\times 10^{2}$
f26	$4.75\times 10^{2}$	$4.69\times 10^{2}$	$4.69\times 10^{2}$	$4.67\times 10^{2}$	$4.73\times 10^{2}$	$4.67\times 10^{2}$
f27	$8.52\times 10^{2}$	$8.62\times 10^{2}$	$8.45\times 10^{2}$	$8.38\times 10^{2}$	$8.22\times 10^{2}$	$8.45\times 10^{2}$
f28	$9.74\times 10^{2}$	$9.43\times 10^{2}$	$1.03\times 10^{3}$	$8.20\times 10^{2}$	$9.33\times 10^{2}$	$1.26\times 10^{3}$

**Table 6:** The running results of the standard deviation of each algorithm.

Functions	PCSCAS1	PCSCAS2	PCSCAS3	PCFDA1	PCFDA2	PCFDA3
f1	$6.53\times 10^{2}$	$5.34\times 10^{2}$	$5.82\times 10^{2}$	$6.67\times 10^{2}$	$6.65\times 10^{2}$	$6.67\times 10^{2}$
f2	$3.60\times 10^{7}$	$3.73\times 10^{7}$	$4.91\times 10^{7}$	$1.57\times 10^{7}$	$2.19\times 10^{7}$	$2.04\times 10^{7}$
f3	$1.50\times 10^{10}$	$1.07\times 10^{10}$	$1.49\times 10^{10}$	$5.02\times 10^{9}$	$5.14\times 10^{9}$	$6.21\times 10^{9}$
f4	$4.61\times 10^{4}$	$5.17\times 10^{4}$	$4.78\times 10^{4}$	$2.46\times 10^{4}$	$2.38\times 10^{4}$	$2.53\times 10^{4}$
f5	$3.98\times 10^{2}$	$2.62\times 10^{2}$	$2.68\times 10^{2}$	$4.42\times 10^{2}$	$4.43\times 10^{2}$	$4.33\times 10^{2}$
f6	$3.67\times 10^{2}$	$3.62\times 10^{2}$	$3.65\times 10^{2}$	$3.73\times 10^{2}$	$3.94\times 10^{2}$	$3.72\times 10^{2}$
f7	$2.74\times 10^{2}$	$2.82\times 10^{2}$	$2.85\times 10^{2}$	$3.07\times 10^{2}$	$2.92\times 10^{2}$	$3.10\times 10^{2}$
f8	$3.26\times 10^{2}$	$3.26\times 10^{2}$	$3.25\times 10^{2}$	$3.26\times 10^{2}$	$3.26\times 10^{2}$	$3.26\times 10^{2}$
f9	$2.70\times 10^{2}$	$2.71\times 10^{2}$	$2.70\times 10^{2}$	$2.71\times 10^{2}$	$2.72\times 10^{2}$	$2.71\times 10^{2}$
f10	$1.71\times 10^{2}$	$1.37\times 10^{2}$	$1.14\times 10^{2}$	$2.15\times 10^{2}$	$2.25\times 10^{2}$	$2.10\times 10^{2}$
f11	$5.68\times 10^{1}$	$3.55\times 10^{1}$	$4.65\times 10^{1}$	$4.99\times 10^{1}$	$6.29\times 10^{1}$	$7.90\times 10^{1}$
f12	$1.11\times 10^{2}$	$5.59\times 10^{1}$	$1.02\times 10^{2}$	$4.03\times 10^{1}$	$2.85\times 10^{1}$	$5.50\times 10^{1}$
f13	$1.28\times 10^{2}$	$8.24\times 10^{1}$	$7.91\times 10^{1}$	$7.37\times 10^{1}$	$7.81\times 10^{1}$	$9.82\times 10^{1}$
f14	$2.70\times 10^{3}$	$3.11\times 10^{3}$	$2.91\times 10^{3}$	$2.51\times 10^{3}$	$2.47\times 10^{3}$	$2.08\times 10^{3}$
f15	$3.04\times 10^{3}$	$3.16\times 10^{3}$	$3.32\times 10^{3}$	$2.66\times 10^{3}$	$2.56\times 10^{3}$	$2.81\times 10^{3}$
f16	$9.74\times 10^{1}$	$9.73\times 10^{1}$	$9.75\times 10^{1}$	$9.72\times 10^{1}$	$9.70\times 10^{1}$	$9.71\times 10^{1}$
f17	$3.81\times 10^{2}$	$3.65\times 10^{2}$	$4.06\times 10^{2}$	$3.02\times 10^{2}$	$3.10\times 10^{2}$	$3.27\times 10^{2}$
f18	$3.92\times 10^{2}$	$3.93\times 10^{2}$	$4.05\times 10^{2}$	$3.70\times 10^{2}$	$3.68\times 10^{2}$	$3.70\times 10^{2}$
f19	$2.62\times 10^{2}$	$2.57\times 10^{2}$	$2.65\times 10^{2}$	$2.53\times 10^{2}$	$2.51\times 10^{2}$	$2.53\times 10^{2}$
f20	$2.95\times 10^{2}$	$2.95\times 10^{2}$	$2.95\times 10^{2}$	$2.95\times 10^{2}$	$2.95\times 10^{2}$	$2.95\times 10^{2}$
f21	$5.28\times 10^{2}$	$5.97\times 10^{2}$	$6.08\times 10^{2}$	$5.23\times 10^{2}$	$5.13\times 10^{2}$	$5.07\times 10^{2}$
f22	$3.44\times 10^{3}$	$3.88\times 10^{3}$	$3.92\times 10^{3}$	$2.91\times 10^{3}$	$3.19\times 10^{3}$	$3.00\times 10^{3}$
f23	$3.76\times 10^{3}$	$3.75\times 10^{3}$	$4.07\times 10^{3}$	$3.60\times 10^{3}$	$3.34\times 10^{3}$	$3.48\times 10^{3}$
f24	$6.30\times 10^{2}$	$6.30\times 10^{2}$	$6.26\times 10^{2}$	$6.25\times 10^{2}$	$6.20\times 10^{2}$	$6.24\times 10^{2}$
f25	$6.83\times 10^{2}$	$6.81\times 10^{2}$	$6.83\times 10^{2}$	$6.75\times 10^{2}$	$6.72\times 10^{2}$	$6.80\times 10^{2}$
f26	$6.84\times 10^{2}$	$6.75\times 10^{2}$	$6.75\times 10^{2}$	$6.72\times 10^{2}$	$6.81\times 10^{2}$	$6.72\times 10^{2}$
f27	$1.23\times 10^{3}$	$1.24\times 10^{3}$	$1.22\times 10^{3}$	$1.21\times 10^{3}$	$1.18\times 10^{3}$	$1.22\times 10^{3}$
f28	$1.44\times 10^{3}$	$1.38\times 10^{3}$	$1.50\times 10^{3}$	$1.46\times 10^{3}$	$1.55\times 10^{3}$	$1.97\times 10^{3}$

**Table 7:** The comparison results between PCFDA1 with three improved SCA algorithms.

Functions	PCSCAS1	PCSCAS2	PCSCAS3
f1	$1.827\times 10^{-4}$	$1.827\times 10^{-4}$	$1.827\times 10^{-4}$
f2	$3.121\times 10^{-2}$	$1.315\times 10^{-3}$	$7.285\times 10^{-3}$
f3	$1.315\times 10^{-3}$	$7.285\times 10^{-3}$	$2.113\times 10^{-2}$
f4	$1.827\times 10^{-4}$	$1.827\times 10^{-4}$	$1.827\times 10^{-4}$
f5	$1.827\times 10^{-4}$	$1.827\times 10^{-4}$	$1.827\times 10^{-4}$
f6	$3.764\times 10^{-2}$	$5.828\times 10^{-4}$	$4.727\times 10^{-2}$
f7	$4.727\times 10^{-2}$	$4.727\times 10^{-2}$	$9.108\times 10^{-3}$
f8	$5.205\times 10^{-1}$	$9.698\times 10^{-1}$	$5.708\times 10^{-1}$
f9	$3.075\times 10^{-2}$	$1.405\times 10^{-2}$	$1.402\times 10^{-2}$
f10	$1.827\times 10^{-4}$	$1.827\times 10^{-4}$	$1.827\times 10^{-4}$
f11	$1.706\times 10^{-3}$	$2.827\times 10^{-3}$	$3.764\times 10^{-2}$
f12	$2.575\times 10^{-2}$	$7.285\times 10^{-3}$	$3.075\times 10^{-2}$
f13	$3.847\times 10^{-2}$	$2.113\times 10^{-2}$	$3.847\times 10^{-2}$
f14	$3.298\times 10^{-4}$	$7.685\times 10^{-4}$	$4.396\times 10^{-4}$
f15	$1.315\times 10^{-3}$	$4.396\times 10^{-4}$	$1.726\times 10^{-2}$
f16	$3.764\times 10^{-2}$	$4.640\times 10^{-2}$	$1.859\times 10^{-2}$
f17	$1.402\times 10^{-2}$	$3.298\times 10^{-4}$	$4.910\times 10^{-2}$
f18	$2.575\times 10^{-2}$	$3.121\times 10^{-2}$	$1.620\times 10^{-2}$
f19	$2.461\times 10^{-4}$	$2.827\times 10^{-3}$	$4.890\times 10^{-2}$
f20	$2.730\times 10^{-1}$	$7.337\times 10^{-1}$	$4.274\times 10^{-1}$
f21	$1.827\times 10^{-4}$	$1.827\times 10^{-4}$	$1.405\times 10^{-2}$
f22	$1.827\times 10^{-4}$	$2.202\times 10^{-3}$	$4.586\times 10^{-3}$
f23	$3.121\times 10^{-2}$	$1.008\times 10^{-3}$	$4.678\times 10^{-2}$
f24	$4.396\times 10^{-4}$	$5.828\times 10^{-4}$	$3.075\times 10^{-2}$
f25	$2.461\times 10^{-4}$	$5.795\times 10^{-3}$	$2.461\times 10^{-4}$
f26	$4.396\times 10^{-4}$	$3.298\times 10^{-4}$	$4.396\times 10^{-4}$
f27	$4.850\times 10^{-2}$	$1.405\times 10^{-2}$	$4.791\times 10^{-2}$
f28	$1.405\times 10^{-2}$	$1.405\times 10^{-2}$	$1.008\times 10^{-3}$

**Table 8:** The comparison results between PCFDA2 with three improved SCA algorithms.

Functions	PCSCAS1	PCSCAS2	PCSCAS3
f1	$1.827\times 10^{-4}$	$1.827\times 10^{-4}$	$1.827\times 10^{-4}$
f2	$2.113\times 10^{-2}$	$1.706\times 10^{-3}$	$7.285\times 10^{-3}$
f3	$4.396\times 10^{-4}$	$3.611\times 10^{-3}$	$2.113\times 10^{-2}$
f4	$1.827\times 10^{-4}$	$3.298\times 10^{-4}$	$1.827\times 10^{-4}$
f5	$1.827\times 10^{-4}$	$1.827\times 10^{-4}$	$1.827\times 10^{-4}$
f6	$3.121\times 10^{-2}$	$7.685\times 10^{-4}$	$4.727\times 10^{-2}$
f7	$1.620\times 10^{-2}$	$1.402\times 10^{-2}$	$9.108\times 10^{-3}$
f8	$6.232\times 10^{-1}$	$9.097\times 10^{-1}$	$5.708\times 10^{-1}$
f9	$4.757\times 10^{-2}$	$3.539\times 10^{-2}$	$1.402\times 10^{-2}$
f10	$1.827\times 10^{-4}$	$1.827\times 10^{-4}$	$1.827\times 10^{-4}$
f11	$1.402\times 10^{-2}$	$1.402\times 10^{-2}$	$3.764\times 10^{-2}$
f12	$4.970\times 10^{-2}$	$1.212\times 10^{-2}$	$3.075\times 10^{-2}$
f13	$3.482\times 10^{-2}$	$2.123\times 10^{-2}$	$3.847\times 10^{-2}$
f14	$7.685\times 10^{-4}$	$3.611\times 10^{-3}$	$4.396\times 10^{-4}$
f15	$9.108\times 10^{-3}$	$1.008\times 10^{-3}$	$1.726\times 10^{-2}$
f16	$4.539\times 10^{-2}$	$3.447\times 10^{-2}$	$1.859\times 10^{-2}$
f17	$1.859\times 10^{-2}$	$9.108\times 10^{-3}$	$4.910\times 10^{-2}$
f18	$3.467\times 10^{-2}$	$4.571\times 10^{-2}$	$1.620\times 10^{-2}$
f19	$7.285\times 10^{-3}$	$4.515\times 10^{-2}$	$3.890\times 10^{-2}$
f20	$1.402\times 10^{-2}$	$2.730\times 10^{-1}$	$4.274\times 10^{-1}$
f21	$1.827\times 10^{-4}$	$1.827\times 10^{-4}$	$1.405\times 10^{-2}$
f22	$1.827\times 10^{-4}$	$7.685\times 10^{-4}$	$4.586\times 10^{-3}$
f23	$3.611\times 10^{-3}$	$4.396\times 10^{-4}$	$4.678\times 10^{-1}$
f24	$4.970\times 10^{-2}$	$4.850\times 10^{-2}$	$3.075\times 10^{-2}$
f25	$4.515\times 10^{-2}$	$4.910\times 10^{-2}$	$2.461\times 10^{-4}$
f26	$1.827\times 10^{-4}$	$5.183\times 10^{-2}$	$5.440\times 10^{-2}$
f27	$4.970\times 10^{-2}$	$3.075\times 10^{-2}$	$4.791\times 10^{-2}$
f28	$1.706\times 10^{-3}$	$1.315\times 10^{-3}$	$1.008\times 10^{-3}$

**Table 9:** The comparison results between PCFDA3 with three improved SCA algorithms.

Functions	PCSCAS1	PCSCAS2	PCSCAS3
f1	$1.827\times 10^{-4}$	$1.827\times 10^{-4}$	$1.827\times 10^{-4}$
f2	$3.791\times 10^{-2}$	$3.447\times 10^{-2}$	$7.285\times 10^{-3}$
f3	$1.212\times 10^{-2}$	$4.274\times 10^{-2}$	$2.113\times 10^{-2}$
f4	$1.827\times 10^{-4}$	$7.685\times 10^{-4}$	$1.827\times 10^{-4}$
f5	$1.827\times 10^{-4}$	$1.827\times 10^{-4}$	$1.827\times 10^{-4}$
f6	$4.640\times 10^{-2}$	$7.685\times 10^{-4}$	$4.727\times 10^{-2}$
f7	$4.623\times 10^{-2}$	$4.890\times 10^{-2}$	$9.108\times 10^{-3}$
f8	$1.212\times 10^{-1}$	$7.566\times 10^{-2}$	$5.708\times 10^{-1}$
f9	$1.620\times 10^{-1}$	$7.566\times 10^{-2}$	$1.402\times 10^{-2}$
f10	$2.202\times 10^{-3}$	$1.008\times 10^{-3}$	$1.827\times 10^{-4}$
f11	$3.764\times 10^{-2}$	$4.515\times 10^{-2}$	$3.764\times 10^{-2}$
f12	$4.623\times 10^{-2}$	$4.890\times 10^{-2}$	$3.075\times 10^{-2}$
f13	$4.727\times 10^{-2}$	$7.566\times 10^{-2}$	$3.847\times 10^{-1}$
f14	$3.611\times 10^{-3}$	$1.402\times 10^{-2}$	$4.396\times 10^{-4}$
f15	$3.121\times 10^{-2}$	$1.315\times 10^{-3}$	$1.726\times 10^{-2}$
f16	$3.847\times 10^{-2}$	$4.910\times 10^{-2}$	$1.859\times 10^{-2}$
f17	$4.890\times 10^{-2}$	$7.685\times 10^{-4}$	$4.910\times 10^{-2}$
f18	$4.586\times 10^{-3}$	$3.764\times 10^{-2}$	$1.620\times 10^{-2}$
f19	$4.539\times 10^{-2}$	$1.212\times 10^{-2}$	$3.890\times 10^{-2}$
f20	$1.008\times 10^{-3}$	$7.285\times 10^{-3}$	$4.274\times 10^{-1}$
f21	$1.827\times 10^{-4}$	$1.827\times 10^{-4}$	$1.405\times 10^{-2}$
f22	$1.133\times 10^{-2}$	$1.041\times 10^{-2}$	$4.586\times 10^{-3}$
f23	$7.285\times 10^{-3}$	$5.828\times 10^{-4}$	$4.678\times 10^{-1}$
f24	$4.890\times 10^{-2}$	$1.859\times 10^{-2}$	$3.075\times 10^{-2}$
f25	$2.461\times 10^{-4}$	$5.795\times 10^{-2}$	$2.461\times 10^{-4}$
f26	$4.586\times 10^{-3}$	$9.108\times 10^{-3}$	$4.396\times 10^{-4}$
f27	$4.734\times 10^{-2}$	$4.521\times 10^{-2}$	$4.791\times 10^{-2}$
f28	$5.708\times 10^{-1}$	$7.337\times 10^{-1}$	$5.101\times 10^{-2}$

**Table 10:** Experimental results of the localization error of different anchor nodes.

Algorithms	Evaluation Indicators	10	20	30	40	50
MCL	Ave	23.6269	16.3017	11.8374	9.7348	8.8307
	Std	2.8153	0.8655	0.5684	0.5097	0.7238
FDA	Ave	20.8774	15.1468	7.5836	5.5885	6.2892
	Std	4.5022	11.0386	4.8461	5.5609	3.6356
cAPSO	Ave	20.2842	15.1823	9.3829	7.2389	5.8412
	Std	2.7114	1.0986	0.8491	0.4829	0.2983
PCFDA1	Ave	19.2934	15.3938	6.2677	5.4326	3.2778
	Std	2.1672	1.0578	0.5648	0.4364	0.5363
PCFDA2	Ave	19.3737	14.3896	6.3897	5.2874	3.9473
	Std	2.6438	1.1724	0.6573	0.5483	0.6372
PCFDA3	Ave	19.2478	15.3851	6.0837	5.3573	3.3732
	Std	2.6327	1.2563	0.5885	0.8356	0.6334

**Table 11:** Experimental results of the localization error of different communication radius.

Algorithms	Evaluation Indicators	20	40	60	80
MCL	Ave	16.8253	11.1635	7.5723	6.6517
	Std	2.2746	0.8523	0.2663	0.4111
FDA	Ave	14.0564	8.5623	4.7753	1.3562
	Std	9.2742	10.6584	4.2358	5.2645
cAPSO	Ave	14.3829	8.5933	4.8321	1.8932
	Std	1.3922	1.0529	0.4721	0.2292
PCFDA1	Ave	7.0317	3.3189	1.5642	0.6523
	Std	1.7834	0.9748	0.5943	0.2984
PCFDA2	Ave	6.9851	3.3451	1.1567	0.6586
	Std	1.8375	0.9732	0.5382	0.4928
PCFDA3	Ave	6.9856	3.7652	1.3654	0.8562
	Std	1.9382	0.9375	0.4878	0.7362

## Data Availability

Not applicable.
